# The Effect of Heat Treatment on the Sensitized Corrosion of the 5383-H116 Al-Mg Alloy

**DOI:** 10.3390/ma10030275

**Published:** 2017-03-09

**Authors:** Ying-Kai Lin, Shing-Hai Wang, Ren-Yu Chen, Tso-Sheng Hsieh, Liren Tsai, Chia-Chin Chiang

**Affiliations:** 1 Department of Mechanical Engineering, National Kaohsiung University of Applied Sciences, Kaohsiung City 807, Taiwan; u8812331@gmail.com (Y.-K.L.); srcx2s904@gmail.com (T.-S.H.); liren@kuas.edu.tw (L.T.); 2 Department of Electrical Engineering, Fortune Institute of Technology, No. 28, Zhixue Rd., Daliao Dist., Kaohsiung City 831, Taiwan; shing-hae_wang@hotmail.com; 3 Naval Shipbuilding Development Center, Navy Command R.O.C., Taipei City 104, Taiwan; renyu.tw@gamil.com

**Keywords:** 5383-H116 Al-Mg alloy, sensitization, NAMLT, β phase-Al_3_Mg_2_

## Abstract

In this study, the effects of heat treatment and sensitized corrosion on the 5383-H116 Al-Mg alloy were investigated for temperatures ranging from 100 to 450 °C. The results show that the heat treatment temperature is the main factor that causes changes to the microstructure and mechanical strength of the 5383-H116 Al-Mg alloy, inducing β-phase (Al_3_Mg_2_) precipitation in the form of a continuous layer along the grain boundaries. Intergranular corrosion was caused by the β-phase of the grain boundary precipitation, and the corrosion susceptibility of the recrystallized structure was significantly higher than the corrosion susceptibility of the recovered structure. According to the conductivity values detected, β-phase precipitation can enhance the 5383-H116 Al-Mg alloy conductivity, with the response due to structural dislocation density being higher than that due to the recrystallized structure. As such, the β-phase precipitation after sensitization is more significant than the β-phase precipitation prior to the sensitization, such that after sensitization, the conductivity rises to a significantly higher level than that exhibited by the recrystallization structure.

## 1. Introduction

The 5XXX series Al-Mg alloy is known to be lightweight, easy to machine, and high in strength, among other attributes [1,2]. Since the 1950s, large amounts of the alloy have been used in the structures of ships due to its excellent corrosion resistance when immersed in seawater [3,4]. Use of the alloy allowed the weights of vessels produced with it to be significantly reduced, an improvement which translated into fuel savings and associated environmental advantages [5]. However, the applications of aluminum and its alloys in this field were far less advanced than those of various steels because of cost and formability issues at room temperature [6]. Marine applications for Al-Mg alloys are mainly divided into the construction of ship frames and outfitting. The aluminum materials used by each shipyard differ according to the ship design and the task requirements. ASTM B928 standards state that the 5086, 5083, 5383, 5456, and 5059 Al-Mg alloys and H116 and H321 Al-Mg tempers are suitable for marine service. H116 temper plates are manufactured and hardened using cold rolling, while H321 temper plates are stabilized by low-temperature heating after the hardening process in order to cause the β-phase to be evenly spread through the Al matrix, thus improving the intergranular corrosion and stripping resistances. The main alloying element in marine Al-Mg alloys is Mg; the strength increases as the amount of Mg increases [7]. Popović [8] proposed that the change in the differential dislocation density during the recovery or recrystallization has little effect on the conductivity, and the main difference is due to the solid solution or precipitation of the β-phase. The primary corrosion problems of Al-Mg alloys, in which the β-phase Al_3_Mg_2_ has a temperature range of 50–200 °C, will gradually result in a sensitization phenomenon due to the formation of β-phase layers at the grain boundaries. In this study, we observed microstructure changes and used the nitric acid mass loss test (NAMLT), known as the ASTM G67-13 NAMLT, to investigate the corrosion characteristics of the 5383-H116 Al-Mg alloy [9].

## 2. Literature and Theory

### 2.1. Al-Mg Alloy Sensitization Characteristics

ASTM B928 defines Al-Mg alloy sensitization as the precipitation of the β-phase at the grain boundaries [10]. When the density of β-phase precipitates increases, the degree of sensitization (DoS) also increases. Under corrosive or stressful conditions, this leads to intergranular corrosion and stress corrosion. In addition, as the DoS within the microstructure cannot be exactly determined, ASTM B928 requires Al-Mg alloy to be subject to the ASTM G67-13 NAMLT [11] to measure the mass loss during corrosion in order to ascertain the DoS. A mass loss of less than 15 mg/cm^2^ is considered not sensitized, a mass loss of between 15 and 25 mg/cm^2^ is considered partially sensitized, and a mass loss of over 25 mg/cm^2^ is considered sensitized.

### 2.2. β-Phase Precipitation Characteristics and Effects on Corrosion

#### 2.2.1. Influence of Mg Content on β-Phase Precipitation

Gupta et al. [12] examined the NAMLT mass loss and β-phase precipitation of Al-Mg alloys with varying Mg contents after sensitization treatment (150 °C/7 days) and found that with a Mg content >4 wt. %, the mass loss increased linearly and the proportion of the β-phase precipitation increased as the Mg content increased. Dix et al. [13] investigated the influence of Mg content on Al-Mg alloy sensitization and stress corrosion and found potential differences between the precipitates and the Al matrix, leading them to propose the Al-Mg alloy anodic dissolution stress corrosion mechanism wherein the stress corrosion sensitivity increases as the Mg content increases. Czyryca et al. [14] tested 5086, 5083, and 5456 Al-Mg alloys for corrosion when soaked in saltwater, when salt-spray tested, and under harbor exposure conditions. The results showed that the degree of corrosion and the Mg content were directly proportional, with the 5086 alloy experiencing the lowest level of corrosion, followed by the 5083 and 5456 alloys.

#### 2.2.2. Influence of Sensitization Temperature and Time on β-Phase Precipitation

Searles et al. [15] pointed out that the sensitization temperatures of 5XXX Al-Mg alloys are between 50–200 °C and that different sensitization temperatures and isothermal holding times cause different amounts of corrosion. In addition, the sensitization temperature range and isothermal holding time are the main factors influencing Al-Mg alloy β-phase precipitation. Oguocha et al. [16] tested the corrosion resistance of 5083-H116 at 80, 100, 150, 175, and 200 °C and at different heating times and found that the 150–200 °C range caused high sensitization, with the most sensitization and corrosion mass loss occurring at 175 °C. They also noted that in a corrosive environment, mass loss increases as the isothermal holding time increases. Jones et al. [17] investigated the corrosion of 5083-H321 at 175 °C with different heating times and concluded that as the heating time increased within the 50–200 °C sensitization range, the β-phase precipitation became more dense and connected and the fissure growth rate increased; thus, the β-phase precipitation density increased and the stress corrosion became more significant. Lim et al. [18] investigated the 5083 Al-Mg alloy intergranular corrosion permeation depth and the relationship between the electrochemical corrosion environment and metallurgy conditions and found that the β-phase corrosion depth and sensitization time were directly proportional and affected by the grain direction, specifically that corrosion permeated following the rolling direction.

## 3. Experiment Setup

This study used 6-mm-thick 5383-H116 Al-Mg alloy plates produced by Alcan incorporation. Prior to the experiment, the composition of the sample was analyzed using a glow discharge spectrometer (GDS) as shown in Table 1.

### 3.1. Heat Treatment Stage

5383-H116 aluminum alloys were cut into cubes (10 mm × 10 mm × 10 mm for optical microscope (OM) and scanning electron microscopy (SEM)) and cuboids (6 mm × 6 mm × 10 mm, for ASTM G-67 mass loss test), and subsequently placed in ovens for isothermal aging experiments. The aging temperatures were set to 25, 100, 150, 200, 250, 300, 350, 400 and 450 °C (the heating rate was about 10 °C/min and the temperature of the ovens was controlled to within 0.5 °C) to produce microstructure changes in the alloys and held there for 30 min before quenching of the alloys was performed in room temperature water. The heat treated test samples then underwent sensitization treatment at 175 °C for 168 h and were then water quenched. After the β-phase precipitated, the samples were analyzed under a microscope, underwent corrosion testing, and were measured for electrical conductivity.

### 3.2. Corrosion Testing

According to ASTM G67, the dimensions of the test specimen were 50 mm × 6 mm × 6 mm, and the specimen was soaked in 5% NaOH solution at 80 °C to remove the surface oxidation and impurities. This was followed by its continuous immersion in HNO_3_ solution at 30 °C for 24 h to calculate the weight loss per unit area (mg/cm^2^), which allowed, in turn, for the evaluation of the sensitivity of the intergranular corrosion.

### 3.3. Measuring Electrical Conductivity

By measuring the electrical conductivity, it is possible to assess the microstructure of the alloy and the changes in β phase precipitation. This study used a Sigmascope SMP10 (Fischer Technology, Inc., Windsor, CT, USA) for electrical conductivity measurement. Calibration was completed before the experiment using the International Annealed Copper Standard (IACS); the unit of this test was % IACS. After calibration, the electrical conductivity of each test strip was measured to investigate the changes in conductivity in alloys manufactured with different heat treatments and different sensitivities.

## 4. Results and Discussion

### 4.1. Microstructure Changes Resulting from the Heat Treatment

**Figure 1:**
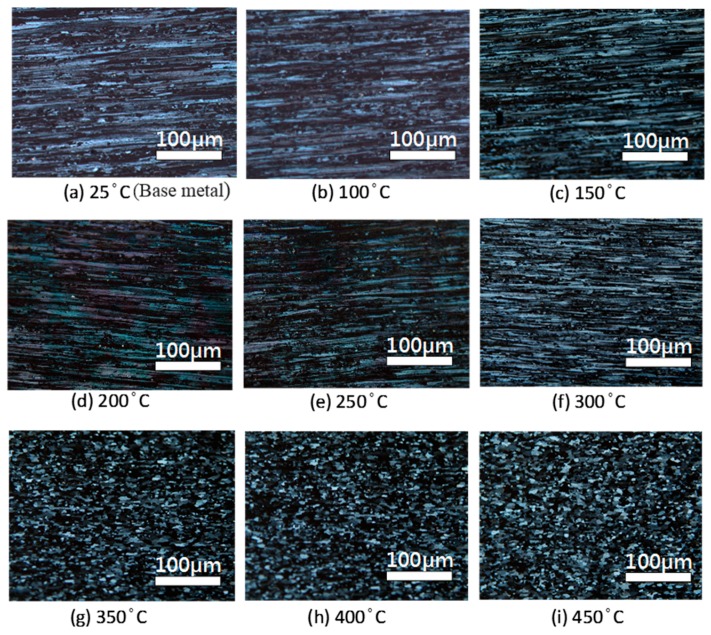
OM images of 5383-H116 Al-Mg alloy at different heat treatment temperatures. (**a**) 25 °C; (**b**) 100 °C; (**c**) 150 °C; (**d**) 200 °C; (**e**) 250 °C; (**f**) 300 °C; (**g**) 350 °C; (**h**) 400 °C; (**i**) 450 °C.

The overall microstructure was similar to the original material during the recovery phase from 25–300 °C and there were no noticeable changes. The grains were still fibrous and distributed in the direction of rolling (Figure 1b–f). When the temperature surpassed the recrystallization temperature, some grains were ready for nucleation and growth. Fibrous grain structures and small recrystallized structures can both be seen in Figure 1f; the dislocations would be eliminated during the recrystallization process and small grains without strain would be nucleated. At a temperature of 350 °C, the microstructure was already made up of strainless, equiaxed grains. At 450 °C, the equiaxed recrystallized structure was maintained, indicating that the high temperature provided sufficient energy to completely release the stored strain energy. The results indicated that suitable heat treatment temperatures are lower than the recovery temperature of 250 °C, as no major changes to the microstructure occurred when the samples were treated at such temperatures. When the temperature was over 300 °C, however, the microstructure recrystallized and the mechanical strength decreased. Heat treatment temperatures of 65–200 °C caused β-phase precipitation and sensitization. In a corrosive environment, this will cause intergranular corrosion and stress corrosion.

### 4.2. Influence of Sensitization on Microstructure and Mechanical Properties

**Figure 2:**
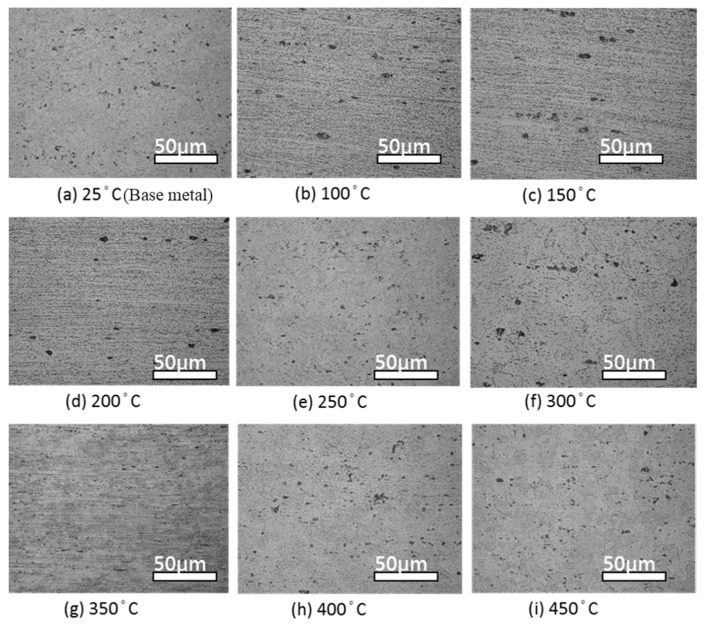
OM images of 5383-H116 Al-Mg alloy β-phase precipitation at different annealing temperatures. (**a**) 25 °C; (**b**) 100 °C; (**c**) 150 °C; (**d**) 200 °C; (**e**) 250 °C; (**f**) 300 °C; (**g**) 350 °C; (**h**) 400 °C; (**i**) 450 °C.

**Figure 3:**
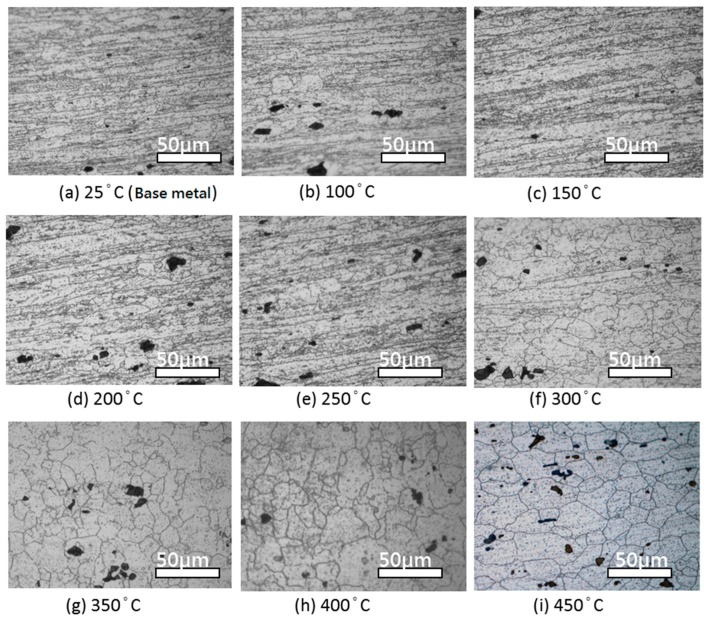
OM images of 5383-H116 Al-Mg alloy β-phase precipitation at different annealing temperatures after 175 °C/168 h sensitization. (**a**) 25 °C; (**b**) 100 °C; (**c**) 150 °C; (**d**) 200 °C; (**e**) 250 °C; (**f**) 300 °C; (**g**) 350 °C; (**h**) 400 °C; (**i**) 450 °C.

After sensitization treatment, different microstructure patterns were produced due to the different heat treatment temperatures. Also, because the β-phase underwent heterogeneous nucleation at high energy regions, the microstructure patterns first precipitated at the grain boundaries, then dislocated, and finally precipitated within the grain. The β-phase precipitation indicated that the sensitization temperature only caused the β-phase to precipitate and did not change the heat treatment structure. Within the recovery temperature range, the sensitized appearance was still fiber-shaped. At a heat treatment temperature greater than the recrystallization range, the sensitized microstructure transformed from partially recrystallized to fully recrystallized. The 5383-H116 Al-Mg alloy β-phase precipitation at 25–450 °C is shown in Figure 2. Figure 3 shows the β-phase precipitations for each heat treatment after sensitization (175 °C/168 h).

**Figure 4:**
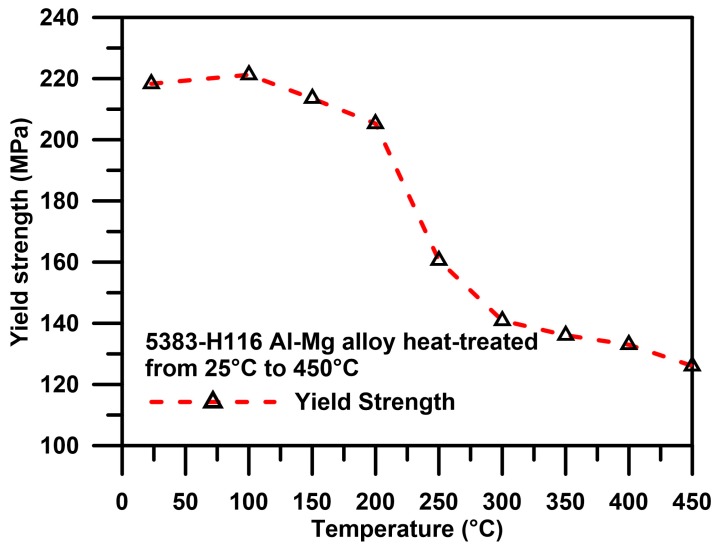
5383-H116 tension test results after different annealing and sensitization treatment.

The results of the stretching tests for the 5383-H116 Al-Mg alloy heat-treated from 25 °C to 450 °C are shown in Figure 4. The Yield Strength (YS) fell by varying degrees as the heat treatment temperature rose. The highest YS was 214 MPa. If the heat treatment temperature was within the recovery period, the loss of tensile strength was negligible; however, as the number of dislocations decreased, creating dislocations with low strain energy, the ductility of the material was increased.

### 4.3. Influence of Heat Treatment and Sensitization Treatment on Intergranular Corrosion Sensitivity

**Figure 5:**
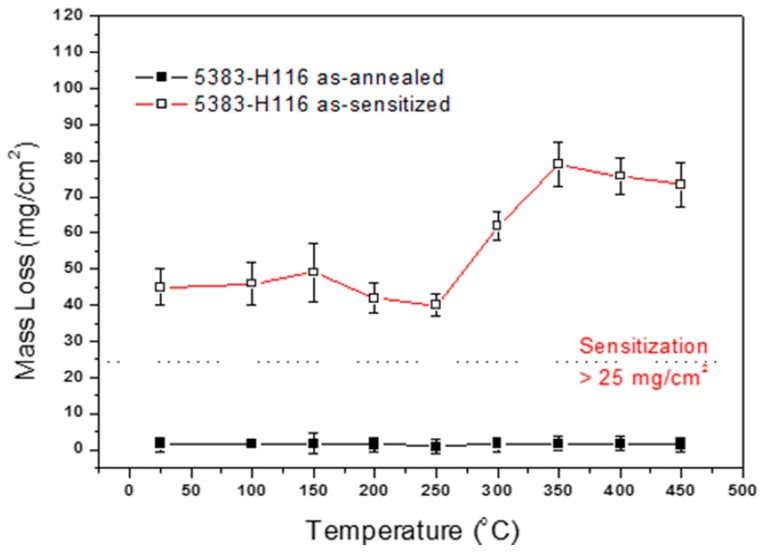
The 5383-H116 NAMLT test results after different heat treatment temperatures and sensitization treatment.

The 5383-H116 Al-Mg alloy test strips heat treated at 25–450 °C for 30 min experienced weight loss within the acceptable parameters (<25 mg/cm^2^), indicating that microstructure transformation did not greatly affect the corrosion resistance. The heat-treated test samples then underwent sensitization treatment at 175 °C for 168 h and were then water-quenched. After the β-phase precipitated, the samples were analyzed under a microscope. Under all heat treatment conditions, the mass loss for the 5383-H116 Al-Mg alloy after sensitization treatment was greater than 25 mg/cm^2^, exceeding the ASTM G67-13 NAMLT standards. Figure 5 shows the 5383-H116 Al-Mg alloy intergranular corrosion sensitivity, with the recrystallized structure clearly greater than the recovery structure. Thus, the β-phase is the main factor that affects intergranular corrosion sensitivity, and the microstructure is the secondary factor that determines intergranular corrosion sensitivity.

The results above show that the β-phase has heterogeneous nucleation at areas of high energy and precipitates first. The β-phase tends to precipitate at grain boundaries regardless of recovery or recrystallization structures. The NAMLT results indicate that the recrystallized structure mass loss is higher than that for recovery structures; therefore, DoS in the recrystallized structure was greater than that in the recovery structure. After the β-phase precipitates and corrodes, the eroded unit volume is greater in recrystallized structures than in recovered structures, leading to greater weight loss in recrystallized structures.

### 4.4. Changes in Electrical Conductivity during the Sensitization Process

**Figure 6:**
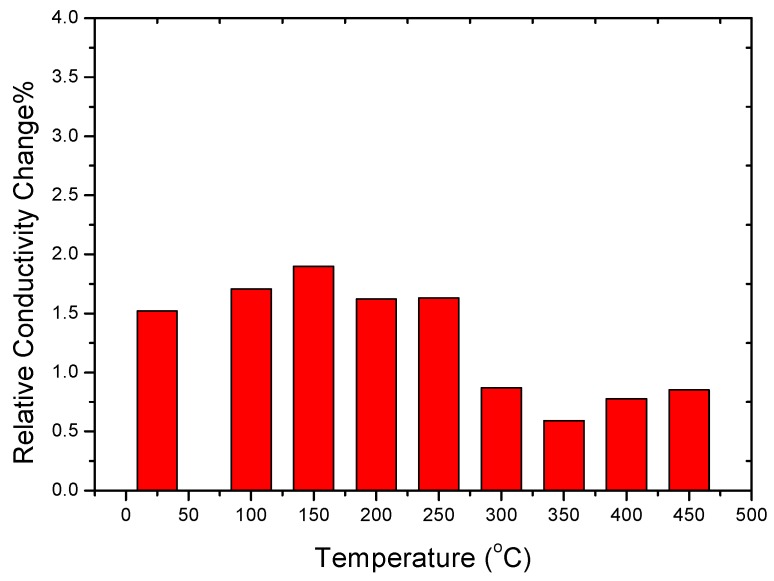
Differences in electrical conductivity in 5383-H116 NAMLT after different heat treatment conditions and sensitization treatment.

The differences in the relative electrical conductivity between 5383-H116 Al-Mg alloys at different heating treatment temperatures before and after sensitization treatment (Δ_σs_) are shown in Figure 6.

Δ_σs_ = (*C*_as-sensitized_ − *C*_as-annealed_)/*C*_as-annealed_ × 100%

where *C*_as-sensitized_ is the electrical conductivity of the heat-treated alloy after sensitization. As shown in the figure, the microstructure transformation did not greatly affect the electrical conductivity, although the electrical conductivity of the heat-treated alloys was increased after sensitization.

The increased electrical conductivity in the recovery structure was greater than in the recrystallized structure because the dislocation density in the recovery structure is greater than the recrystallized structure as the β-phase undergoes heterogeneous nucleation at dislocation areas and the dislocation tube transfer effect diffuses the Mg, i.e., a higher dislocation density increases the rate and amount of β-phase precipitation. Mulazimoglu et al. [19] also pointed out that the increase in the Mg content will reduce the conductivity of the alloy due to a large number of supersaturated Mg atoms being dissolved in the aluminum base, which causes damage to the original lattice arrangement and, thus, significantly hinders its free electron path, thereby reducing the conductivity. According to the Al-Mg binary phase diagram, the β-phase precipitation can be a solution in aluminum by annealing the alloys at elevated temperatures exceeding 250 °C.

### 4.5. NAMLT Corrosion

**Figure 7:**
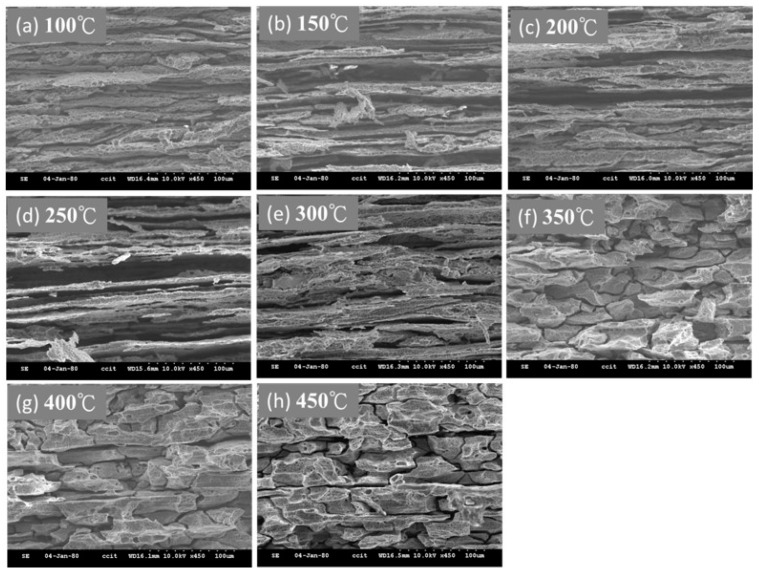
SEM images of corrosion in 5383-H116 after different heat treatment conditions and sensitization treatment. (**a**) 100 °C; (**b**) 150 °C; (**c**) 200 °C; (**d**) 250 °C; (**e**) 300 °C; (**f**) 350 °C; (**g**) 400 °C; (**h**) 450 °C.

Continuous β-phase precipitation provides a pathway for corrosion transmission. The appearance of SEM images of corrosion in 5383-H116 after different heat treatment conditions and sensitization treatment is shown in Figure 7. The corrosion of the 5383-H116 Al-Mg alloy clearly shows that the post-heat treatment microstructure is correlated with the differences in corrosion. Corrosion in the recovery structures was mainly fiber-like in appearance. Corrosion in partly recrystallized structures was partly fiber-like and partly granular in appearance. Finally, corrosion in the fully recrystallized structures was completely granular.

## 5. Conclusions

This study elucidates how heat treatment affects the mechanical properties and sensitization of 5383-H116 aluminum alloys. The following conclusions are drawn from the above analysis.

1. The heat treatment temperature is an important factor affecting 5383-H116 Al-Mg alloy microstructure changes. When the treatment temperature exceeds 300 °C, the structure begins to recrystallize, which decreases the mechanical strength.
2. The DoS of the recrystallized structure is bigger than that of the recovery structure; therefore, in the same sensitized environment, the recrystallized structure will be more susceptible to intergranular corrosion than the recovery structure.
3. The β-phase precipitation helps increase the electrical conductivity, especially in the recovery structure due to the higher dislocation density. In addition, the electrical conductivity after sensitization is markedly increased and can be employed as indirect evidence of the variation of sensitization.

## Figures and Tables

**Table 1:** The components of 5383-H116 Al-Mg alloy (wt. %).

Element	Si	Fe	Cu	Mn	Mg	Cr	Zn	Ti	Al
5383-H116	0.07	0.21	0.09	0.81	4.70	0.08	0.08	0.02	remainder
